# Pregnancy after in vitro fertilization in an elderly primigravida with Shereshevsky-Turner Syndrome: A case report

**DOI:** 10.18502/ijrm.v21i1.12670

**Published:** 2023-02-08

**Authors:** Perizat Saparbaevna Sadykova, Sholpan Kuanyshbekovna Sarmuldayeva, Galina Kalievna Kausova, Elvira Shukenova, Linas Rovas

**Affiliations:** ^1^Medicine Department, Al-Farabi Kazakh National University, Almaty, Kazakhstan.; ^2^Kazakhstan's Medical University, Almaty, Kazakstan.; ^3^Obstetrics Department Maternity Hospital, Almaty, Kazakhstan.; ^4^Klaipeda University Hospital, Klaipeda University, Lithuania.

**Keywords:** Turner syndrome, Pregnancy, Infertility, Hypogonadism, Progesterone.

## Abstract

**Background:**

Shereshevsky-Turner Syndrome is a chromosomal condition that affects females owing to full or partial missing of X-monosomy in all or part of the body's cells. Shereshevsky-Turner Syndrome is characterized by severe hormonal disorders and defects of the cardiovascular and urinary systems. With the advent of assisted reproductive technology (ART), pregnancy has become more accessible for this group of cases, often with donor eggs. In the available literature, it was not possible to find exact information during the time of selection of progestogen support, the duration of the appointment, and the term of withdrawal.

**Case presentation:**

This is the case of a 36-yr-old primigravid woman suffering from STs, mosaic karyotype comprising of 3 clones: 45X (69), 46XX (23), 47XXX (8), and 1000 interphase nuclei. In this case, we left high-maintenance doses of progesterone due to the application of ART and concomitant extragenital pathology, leading to a decrease in all functions of the placenta, including the endocrine. The woman was monitored before, during, and after the pregnancy. She was delivered at 37 wk and 6 days of gestation.

**Conclusion:**

ART increases the possibility of having a pregnancy and gestation in cases with a wide variety of genital and extragenital pathologies.

## 1. Introduction

Shereshevsky-Turner Syndrome (STs) is a chromosomal disorder in females caused by the complete absence or partial absence of monosomy X in all or part of the body's cells. This chromosomal disease occurs at a frequency of 1:2000-1:2500 for baby girls. It is known that 95-98% of women with STs are infertile and incapable of independently conceiving and having a pregnancy without medical help (1, 2). During natural pregnancy, women with STs have a high risk of miscarriages, and the baby born can face the risk of developing congenital malformations. Infertility is the most common problem with ST individuals. According to a study (3), 29% of ST-conceived pregnancies end in spontaneous abortion, 7% in perinatal fetal death, and 20% in the birth of children with developmental defects or abnormalities. In recent years, in vitro fertilization (IVF) method has been developed in clinical practice, increasing the chances of having children with STs significantly. Given the pronounced congenital hypogonadism, pregnancy and childbirth in these women can only be supported by correct and timely hormone replacement therapy (4). The available studies confirm isolated cases of pregnancy in women with STs (5). However, all of them ended in premature birth. With assisted reproductive technology (ART), pregnancy has become more accessible for this group of women, often with donor eggs. Doctors should bring such a woman to full term or the term of a viable fetus. The management of pregnancy in people with IVF with STs is even more difficult due to a large number of somatic pathologies and their hypogonadotropic hypogonadism.

The purpose of this study is to describe the gestation, management, delivery, and breastfeeding in infertile women with a variety of genital and extragenital pathologies. Some issues are quite complex and interesting and require further study.

## 2. Case Presentation

We examined the case of a 36-yr-old primigravid woman with STs. She had a mosaic karyotype with 3 clones: 45X (69), 46XX (23), 47XXX (8), and 1000 interphase nuclei. Karyotype corresponds to STs. The morphotype is characteristic of the syndrome: short stature, obesity, low-hair growth, epicanthus, lymphedema, inverted nipples, and the curvature of the lower leg bones. Pregnancy was done by IVF with a donor egg, height 155 cm, weight 71.3 kg, and body mass index of 29.7 on registration. She was examined by the following specialists: an endocrinologist, cardiologist, geneticist, hematologist, urologist, and therapist. The endocrinologist diagnosed STs hypothyroidism and prescribed eutirox 75 mg once a day for a long time. The ultrasound of the thyroid gland showed a colloid cyst of small size, 0.55 cm. The cardiologist diagnosed chronic arterial hypertension of the first degree. Methyldopa was prescribed 250 mg once a day from the gestational age of 10 wk. During echocardiography, the aorta remained unchanged and not dilated; the valves were intact; the heart cavities were not violated; the systolic function was not impaired, and the diastolic function was type 1 impaired. In addition to STs, the geneticist diagnosed 2 gene polymorphisms: identified homozygous mutations in the fibrinolysis system gene, and a heterozygous mutation in the gene regulating the accumulation of homocysteine. The consequence of these mutations might be associated with a decrease in the fibrinolytic activity of the blood and the accumulation of homocysteine in the blood. After genetic analysis, the hematologist diagnosed congenital thrombophilia. A violation of folate metabolism was identified.

Prescription: folic acid 800 gr, low-molecular weight heparin (LMWH) 0.4 ml subcutaneously 1 time per day under D-dimer control, acetylsalicylic acid 75 mg per day in the evening until 36 wk of pregnancy. The urologist diagnosed for chronic pyelonephritis in remission. Congenital malformations of the urinary system were not detected during the ultrasound study; however, a culture of urine revealed bacteriuria, which was treated with phosphomycin. In addition, phytourinary antiseptics were permanently assigned. The woman accepted calcium preparations of 1.0 gr to prevent hypertensive complications during the 38 ${}^{\text{th}}$ wk of pregnancy. When carrying out an ultrasound of the abdominal organs, the following conclusions were obtained: intestinal pneumatosis, moderate reactive changes in the liver and pancreas, signs of chronic cholecystitis, the expansion of the renal sinus in both kidneys, and dilatation of the cups on the left. However, severe extragenital pathology has not been identified, as reflected in the relatively favorable course of pregnancy. Before pregnancy, hormone therapy was conducted using estradiol and micronized progesterone. After the embryo transfer, a standard supporting therapy of estradiol 1.5 mg was assigned, along with micronized progesterone 300 mg, 200 mg, 300 mg, and progesterone oil solution 2.5 ml intramuscularly. When a heartbeat occurs, estradiol is canceled. Table I presents an illustration of the drugs taken by a pregnant woman during pregnancy.

Thus, the woman took:

• From the time of transfer to 6-7 wk, Estradiol 1.5 mg.

• Progesterone 2.5 ml in the form of an oil solution from the moment of transfer to 20 wk.

• Micronized progesterone from the moment of transfer to 36 wk was given. 800 mg micronized progesterone every day for up to 20 wk. and 700 mg after 20 wk. Progesterone stayed at the values of 49.38-64.56-61.08-58.8 ng/ml. At 30 wk, 500 mg was given in 2 doses; however, at 35 wk one evening dose for 7 days was canceled.

• Under LMWH, the dose was maintained once a day under the control of the D-dimer. Because of the premeditated training, the transfer is canceled and resumed when the ultrasound confirms a heartbeat. Upon receipt of the values of the D-dimer 0.178 mg/l-0.162 mg/l (at a rate of 0.23 mg/l). LMWH was canceled 48 hr before the delivery, and continued after the operation daily during stay in the hospital. When discharged from the hospital, a normal hemostasiogram LMWH is received up to 42 days after childbirth.

• From 10-36 wk, acetylsalicylic acid 75 mg was prescribed daily in the evening.

• Calcium preparations, 1.0 gr daily, from 10-38 wk.

• Phosphomycin once in 14 wk.

• Phytourinary antiseptics with courses of 2-3 wk and interruptions of 2-3 wk from 16 wk before childbirth.

• Methyldopa 250 mg once daily for 10 wk prior to delivery.

• Eutirox 75 mg for a long time.

Ultrasound examinations were conducted according to the protocols for diagnosing chromosomal abnormalities (at 13 wk), congenital malformations of the fetus (at 20 wk), and at 16, 20, 24, and 32 wk for cervicometry. At 20 wk, low placentation was diagnosed (3.7 from the internal os). At 32 wk, this diagnosis was withdrawn. Considering IVF, the underlying pathology, the fetus was monitored for chronic arterial hypertension, congenital thrombophilia, and Doppler measurements at 26-27, 30-31, and 32-33 wk, without disturbing the uteroplacental and fetal-placental blood flow. Cardiotocography (CTG) was performed at 34-35 and 37 wk, with no pathology. During pregnancy, the woman gained 18 kg of weight. Blood pressure throughout the observation period remained stable at 110/70-120/80 mm Hg (while taking methyldopa 250 mg once a day from the 10 ${}^{\text{th}}$ wk). An increase in blood pressure was noted at 35 and 37 wk of gestation, up to 140/90 mm Hg. Lower extremity edema was observed between wk 32 and 33. However, with the lymphedema characteristic of these women, stable blood pressure and the absence of proteinuria were noted. Thus, a drinking regimen and phytouroantiseptics to increase urine filtration were recommended.

In clinical analyses such as complete blood count, urinalysis, and biochemical blood tests, pathology was not revealed. During the entire observation period, the woman was constantly disturbed by vomiting on an empty stomach in the morning. From the 20 ${}^{\text{th}}$ wk, insomnia was observed due to active fetal movement at night. From the outcomes of the therapist, ultrasound of the abdominal organs, and biochemical blood tests, urinalysis did not reveal any pathology, and no action was taken. In addition, other methods that examined the state of the fetus, such as Doppler CTG, did not reveal any pathology. In the 37 ${}^{\text{th}}$ wk of gestation, an increase in edema syndrome and systolic blood pressure of 150-160 mm Hg were observed. The patient was hospitalized in a third-level obstetric facility and delivered by cesarean section routinely at 38 wk. Indications for a planned cesarean section included pregnancy, IVF, severe pre-eclampsia, and lack of preparation of the birth canal. The girl was delivered at 2496 gr, a 48 cm increase, with Apgar scores of 7-8. In the postpartum period, on day 2 came colostrum. However, the child received solid foods in insufficient quantities. After discharge, breastfeeding continued, but pediatricians recommended the lure. Breastfeeding lasted approximately one month. Then, the child is entirely shifted to the mixture. Hormone replacement therapy continued for 42 days after birth. At that moment, the woman received estrogen-progestin replacement therapy to reduce her body mass index by 24 kg/m ${}^{2}$ .

**Table 1:** Presents the diagram of the drugs taken by a case with STs during pregnancy


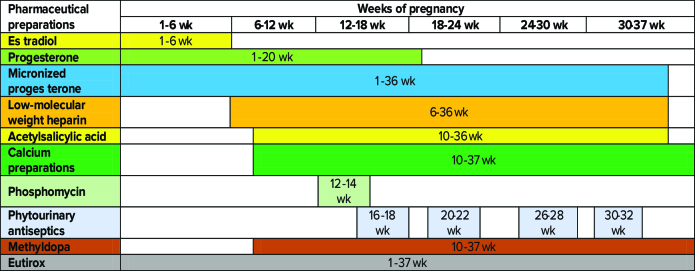

### Ethical considerations

The pregnant woman gave informed consent to conduct this study.

## 3. Discussion

We report the case of a woman with STs, managed by a multidisciplinary team, who achieved pregnancy and successful delivery. Pregnancy in a pregnant woman with STs is associated with a high risk of miscarriage, hypertensive complications, and perinatal and maternal mortality. However, such pregnant women are of high interest to the physician given current punitive medicine. In this clinical case, the possibility of prolonging pregnancy to term is associated with mosaic chromosomes, the absence of severe extragenital pathology, timely prevention, and the identification of concomitant diseases such as thrombophilia, chronic arterial hypertension, hypothyroidism, chronic pyelonephritis, and chronic cholecystitis. Correction of somatic pathology was conducted in conjunction with related specialists, which was simple to carry out. However, hormone therapy, dosage, duration of the prescription, and withdrawal period were complicated since the woman was an IVF pregnancy and had concomitant hypergonadotropic hypogonadism. The great hope for prolonged pregnancy is inspired because the placenta performs the main endocrine function during pregnancy, which begins to form at 8 wk and ends by 16-20 wk of pregnancy (6). However, we withdrew high-maintenance doses of progesterone because of the use of ART and the concomitant extragenital pathology, which led to a decrease in all functions of the placenta, including the endocrine (6). We carried out a monthly determination of the progesterone level, considering the desires and financial capabilities of the woman. We believe this is an optional study because, in our case, the change in the progesterone level does not correlate with a gradual decrease in doses of progesterone administered from outside. However, it is difficult to draw conclusions based on a single clinical example. The issues of delivery in such cases are very interesting. In our case, a question arose about the possibility of spontaneous childbirth. However, with the development of hypertensive pregnancy complications, the doctors' fear of losing a child during childbirth did not come true. However, the question of the delivery method is very controversial, given the level of production of hormones that control the activities of delivery (7, 8). The birth process is likely possible in such cases since the oxytocin produced in the pituitary gland will affect it in any case. Considering hypogonadism, it is impossible to predict how the uterus and cervix will respond. Although with timely hormonal correction, starting from adolescence, a completely adequate response is possible, which is a full-fledged birth process with the birth of a healthy child. The same questions arise with breastfeeding in such women. How long is breastfeeding possible? Is it worth examining and stimulating? Full-fledged breastfeeding within a year and a half after childbirth is recorded. In our case, this cannot happen, despite the woman's desire, which, in all likelihood, does not depend on the underlying pathology. In addition to hormonal support, one of the major problems was congenital thrombophilia, carried out in this case against the background of LMWH with a satisfactory fetal condition. Despite the absence of pathology during Dopplerometry, ultrasound, and CTG, a fetus was born with minor signs of fetal growth retardation syndrome and a good Apgar score. Thus, ART expands the possibilities of having pregnancy and gestation in women with various genital and extragenital pathologies. However, childbearing, maintenance, delivery, and breastfeeding questions for such women are quite complex and interesting, and require further study. The strength of this article is that we very consistently “step by step” describe what treatment (dosages) the pregnant woman received. And it probably would be of interest to other clinicians. We have shown that even in countries with reduced income, it is possible to conceive and give birth successfully with this pathology.

##  Conflict of Interest

The authors declare that there is no conflict of interest.
